# Sociodemographic and clinical characteristics of employees on long-term sick leave

**DOI:** 10.1192/j.eurpsy.2023.1833

**Published:** 2023-07-19

**Authors:** S. Brahim, W. Bouali, S. Khouadja, M. Kacem, R. Ben Soussia, S. Younes, L. Zarrouk

**Affiliations:** psychiatry, Hôpital taher sfar, Mahdia, Tunisia

## Abstract

**Introduction:**

Mental health in the workplace is a rapidly developing field of research, which involves the well-being of the individual on a psychological and social level. However, this balance can be suddenly disrupted and can have such a repercussion that the individual finds himself unable to do his job. In this case, he can benefit from a work stoppage called long term sick leave, governed for the public sector, by the decree number 59-239 of August 24, 1959.

**Objectives:**

To study the socio-demographic and clinical profile of public sector employees who have been on long-term sick leave and examined as part of the assessment of their ability to work.

**Methods:**

It is a retrospective study that focused on all public sector employees on long-term sick leave, examined as part of the evaluation of their ability to work in the psychiatric service CHU MAHDIA during the period from January 2013 to April 2014.

**Results:**

We collected 73 patients. The mean age at the time of the examination was 51.1 years. There was a clear female predominance 67% and the sex ratio was 0.48. The vast majority were married (71%), of average socioeconomic status (52%) and high school level (43%). Most of the patients (67%) were from the Ministry of Public Health, followed by the Ministry of Education with 26% of the study population. The average length of service was 20.4 years with extremes between 3 and 36 years. The average length of leave was 13 months. The most frequent diagnosis of the prescribing physician was adaptation disorder (41%), major depressive disorder (27%). Somatic comorbidity was found in 38% of cases, dominated by hypertension, diabetes and cervicarthritis in 50%, 28% and 18% respectively. At the end of the leave, 82% of the patients were able to return to work. For the other patients, a professional reclassification was necessary.

**Conclusions:**

Long-term sick leave has a heavy economic burden for society and serious socio-economic and psychological repercussions on the patient. Hence the interest in identifying vulnerable subjects and jobs at risk in order to prevent the occurrence of psychopathological disorders.

**Disclosure of Interest:**

None Declared

